# The impact of social interaction on abstract concepts

**DOI:** 10.3758/s13423-026-02941-4

**Published:** 2026-06-18

**Authors:** Daria Goriachun, Jonathan Mirault, Johannes C. Ziegler, Kristof Strijkers

**Affiliations:** 1 Aix-Marseille Univ, CNRS, LPL (UMR 7309), Aix-en-Provence, France; 2 Aix-Marseille Univ, Pôle pilote AMPIRIC, INSPÉ, Marseille, France; 3 Aix-Marseille Univ, CNRS, CRPN (UMR 7077), Marseille, France

**Keywords:** Language, Grounded cognition, Abstract concepts, Social cognition

## Abstract

Social interaction is argued to play a crucial role for grounding abstract knowledge, yet empirical evidence is scarce. To investigate whether social interaction affects the processing of abstract words, 60 French speakers semantically categorized the degree of socialness and concreteness of words either individually or in dyads. When judging the socialness of words, dyads showed a processing advantage for abstract social words that was absent in individual performance. In contrast, when judging the concreteness of words, dyads were less accurate for abstract social words than individuals, confirming that social interaction renders these words more concrete. Dividing the dyads by their level of social engagement based on gaze patterns revealed that the effects were driven by the more cooperative dyads. The findings demonstrate that language processing is distinct between interactive and isolated contexts, where social interaction and engagement dynamically shape the interpretation of abstract concepts.

**Supplementary Information:**

The online version contains supplementary material available at https://doi.org/10.3758/s13423-026-02941-4.

## Introduction

How humans learn and represent abstract concepts remains a central and challenging question for theories of language and cognition. Theories of embodied cognition argue that abstract concepts, like concrete ones, are grounded in sensorimotor experiences (Glenberg et al., 2008; Pecher et al., 2011; Pulvermüller, 2013). However, while there is extensive evidence that concrete concepts, such as objects and actions, activate sensorimotor systems (Connell et al., 2012; Glenberg & Kaschak, 2002; Glenberg et al., 2008; Hauk et al., 2004; Kaschak et al., 2005; Mathôt et al., 2017; Meteyard et al., 2007; Wellsby et al., 2011; Zwaan et al., 2002; Zwaan & Taylor, 2006; but see Morey et al., 2022), much less is known about how abstract concepts are grounded. Indeed, behavioral data consistently reveal differences in the processing of abstract and concrete stimuli (Jessen et al., 2000). For example, the well-documented concreteness effect shows that concrete words have a processing advantage over abstract words. This advantage has been attributed to dual coding: Concrete words engage both verbal and nonverbal systems, whereas abstract words rely primarily on verbal processing (Paivio, 1971, 1990). Supporting this view, neuroimaging studies regularly report greater left-hemisphere activation for abstract words and a more bilateral activation pattern for concrete words (Binder et al., 2005; Fliessbach et al., 2006; Jessen et al., 2000; Perani et al., 1999).

Given the limitations of purely sensorimotor accounts, recent frameworks have proposed a multiple representation view. They emphasize that abstract meaning is shaped not only by sensorimotor experience but also by social, emotional, linguistic, and metacognitive knowledge (Banks et al., 2023; Barsalou et al., 2018; Borghi et al., 2019, 2025; Conca et al., 2021). Despite growing interest in how multiple representations contribute to the grounding of abstract concepts, empirical evidence remains scarce and findings are mixed, particularly regarding social experience (Diveica et al., 2023, 2024; Goriachun et al., 2025). This issue is especially important given that language is, at its core, a social activity. To address this gap, the present study aims to provide evidence on the role of social experience in the grounding of abstract concepts.

We distinguish between two key notions of social experience. “Socialness” refers to a semantic dimension particularly relevant for abstract concepts, which evoke richer social associations (Diveica et al., 2023; Pexman et al., 2023). In contrast, “social metacognition” emphasizes the role of social context and interaction in the processing and acquisition of abstract concepts (Borghi et al., 2021, 2025).

Behavioral studies have examined socialness facilitates word recognition in various psycholinguistic tasks (Diveica et al., 2023, 2024; Goriachun et al., 2025). It has been shown to facilitate word processing in lexical decision tasks (Diveica et al., 2023; Goriachun et al., 2025), as well as in semantic decision, recognition memory, and syntactic classification tasks (Diveica et al., 2024). However, it remains unclear how socialness interacts with concreteness, and whether its effects are stronger for abstract words. Moreover, findings suggest that these effects may be mainly driven by task-specific strategies rather than reflecting the social grounding of abstract (or concrete) knowledge per se (Goriachun et al., 2025).

Given the limited evidence for socialness as an inherent grounding mechanism, it is possible that social interaction itself is necessary to observe the social grounding of language. Research on social metacognition suggests that, in interactive settings, abstract concepts evoke interactional dynamics that differ markedly from those elicited by concrete ones. For instance, Fini et al. (2021) found that participants requested more help when attempting to identify abstract concepts in a concept guessing task, indicating a greater reliance on others (see also Zdrazilova et al., 2018). Similarly, Villani et al. (2022) showed that abstract concepts elicited more turn-taking and interactive exchanges in a simulated conversation task, whereas concrete concepts yielded shorter and more straightforward responses. Together, these findings suggest that abstract concepts rely more on social engagement (Borghi et al., 2022). Communicating abstract ideas may require increased monitoring of one’s own and others’ mental states to construct aligned representations (Banks et al., 2023; Gandolfi et al., 2023; Mazzuca et al., 2025; Villani et al., 2022).

Notwithstanding, strong empirical evidence that abstract concepts could be grounded through social interaction is still lacking. This may be because most studies have investigated language processing in isolated, individual settings. Consequently, one possible reason for the absence of clear effects of socialness in the processing of abstract words is that classical psycholinguistic tasks do not incorporate socially situated context. A key step forward, therefore, is to investigate the grounding of abstract concepts in socially relevant settings. This becomes especially important in the light of recent findings showing that language processing in dyads differ markedly from processing in isolation (Kerr et al., 2025). Therefore, the present study aims to explore how abstract concepts, especially those related to social experience, are processed in a joint task setting.

## The present study

The present experiment builds on a study on the role of socialness in the processing of abstract words (Goriachun et al., 2025) by using the same semantic categorization tasks embedded in a novel joint paradigm, in which participants must reach agreement to successfully perform the task. This design allows us to examine the two key social dimensions: socialness as a semantic property of words and social metacognition as the interactive context in which the task is performed.

As in Goriachun et al. (2025), the study consisted of two semantic categorization tasks:

1. A Socialness Categorization Task, in which words were classified as social or nonsocial, tapping explicitly into socialness; and
2. A Concreteness Categorization Task, where the same words were classified as abstract or concrete, thereby assessing socialness more implicitly.

**Fig. 1:**
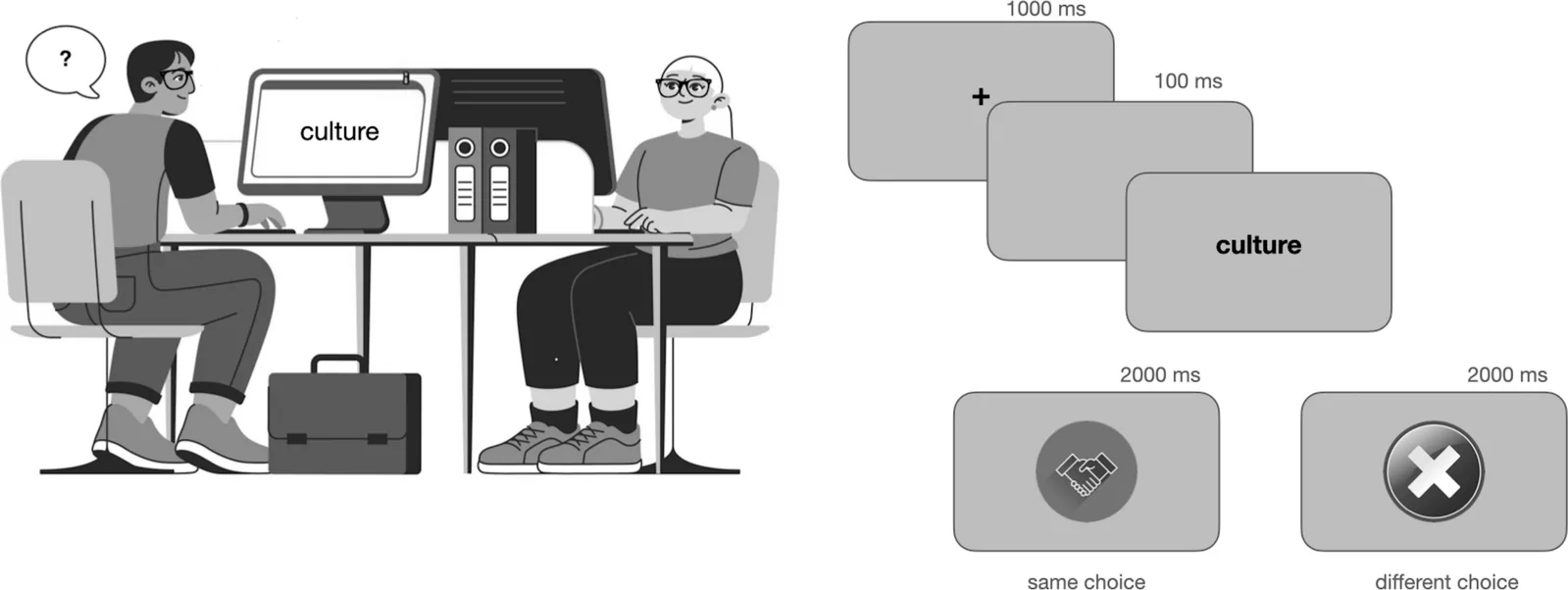
Illustration of the experimental setup and the procedure (dual setting)

Crucially, each task was performed under two conditions:

1. A dual condition, in which two participants performed the task jointly (Fig. 1), and
2. An individual condition, in which participants performed the same tasks individually. In the dual condition, we recorded gaze fixations as an index of their nonverbal interaction in the joint task.

Because the previous study using the same tasks in an individual setting did not reveal significant effects of socialness on the processing of abstract words (Goriachun et al., 2025), we expected to replicate this null effect in the individual condition. In contrast, if the interactive setting enhances the processing of abstract words, we predicted a facilitatory effect on the processing of abstract social words in the dual condition, both when socialness is explicitly relevant to the task (Socialness Categorization Task), and when it is implicit (Concreteness Categorization Task).

## Methods

### Participants

A total of 60 French native speakers were recruited. Of these, 40 participants were assigned to the dual condition, forming 20 dyads who completed the semantic categorization tasks together. Participants were between 18 and 40 years old (*M*_age_ = 21.95 years, *SD*_age_ = 3.99), reported no neurological or language disorders and had normal vision. Participant recruitment did not specify whether individuals were acquainted with one another, as the effect of interpersonal familiarity was not a variable of interest in the present study. Consequently, pairing was left uncontrolled and effectively random. Remaining 20 French native speakers participated in the individual semantic categorization tasks. All participants were aged between 18 and 40 years old (*M*_age_ = 24.8, *SD*_age_ = 3.65). Each participant received 10 euros per session.

### Stimuli

The stimulus set comprised 100 French nouns (see Table 1). Words were selected based on concreteness norms from Brysbaert et al. (2014) and socialness ratings from Diveica et al. (2023). Stimuli were initially chosen in English and subsequently translated into French. The set included four categories of 25 words each: abstract social (e.g., *soutien, culture*; ENG: *guidance, culture*), abstract nonsocial (e.g., *effet, option*; ENG: *effect, option*), concrete social (e.g., *parade, voisin*; ENG: *parade, neighbour),* and concrete nonsocial (e.g., *garage, bracelet*; ENG: *garage, bracelet*). Stimuli were matched for frequency, length, and valence (New et al., 2004). Concreteness ratings for concrete words were above 4, and for abstract words below 3, on a scale from 1 (*very abstract*) to 5 (*very concrete*). Socialness ratings for social words were above 5.5, and for nonsocial words below 3.5, on a scale from 1 (*very nonsocial*) to 7 (*very social*). The same stimulus set was used for both tasks in joint and individual settings.

**Table 1:** Mean lexical and semantic properties of word stimuli for each stimulus type

	Abstract social	Concrete social	Abstract nonsocial	Concrete nonsocial
Concreteness	2.34 (0.51)	4.47 (0.28)	2.5 (0.37)	4.88 (0.11)
Socialness	6.23 (0.26)	5.98 (0.29)	2.29 (0.33)	2.02 (0.7)
Valence	5.25 (1.68)	5.92 (0.72)	5.3 (0.77)	5.51 (1.05)
Length	7.92 (1.68)	7.92 (1.44)	7.4 (1.83)	7.24 (1.42)
Frequency	18.79 (15.59)	17.9 (17)	18 (23.3)	17.56 (13.35)

Values in parentheses represent standard deviations

### Procedure

The stimuli were presented using Labvanced^1^ open-source software running offline in the laboratory. All participants read and accepted a consent form, read the instructions, and performed 10 training trials. The task consisted of two parts. In the Socialness Categorization Task (SCT), participants were required to indicate if the word on the screen was social or nonsocial. In the Concreteness Categorization Task (CCT), participants were asked to judge whether the word on the screen was concrete or abstract. The order of the tasks was counterbalanced across participants. In the dual condition, participants performed the tasks in pairs seated face-to-face in the experimental room (see Fig. 1). Each participant viewed the stimuli presented at the center of their own screen and responded using designated keys on a keyboard. Half of the participants responded to “social” (“concrete”) words with their right hand, and the other half with their left hand. Participants were instructed to respond to each word without communicating verbally with their partner. After each trial, visual feedback indicated whether both participants had made the same choice. In the individual condition, participants performed the same tasks on the same stimuli individually. The procedure was identical to that of the joint task except that no partner feedback was provided. Each participant saw a total of 200 words across the two tasks. The trials were divided into two blocks of 50 items with short breaks between them. The instructions used were adapted from those used in Goriachun et al. (2025) and are provided in the Supplementary Materials.

During the experiment, reaction times (RTs) and error rates were recorded for each participant, along with an agreement rate in the joint tasks. Eye and head movements were recorded using Tobii Glasses 3 eye-tracking glasses, which also captured video and audio throughout the entire experimental session in the dual condition. The setup was arranged so that participants could clearly see their partner’s face but not their response keys.

### Data cleaning and analysis

Data cleaning procedures for both the dual and individual conditions followed the approach described in Goriachun et al. (2025). First, all trials with missing data were excluded. Second, each participant’s individual accuracy was checked, and all participants exceeded the 70% accuracy threshold established for this experiment. Third, word-level accuracy was checked, resulting in the exclusion of one word with an accuracy rate below chance level (i.e., 50%).

Outliers were removed using the interquartile range method (IQR) that was calculated as the difference between third (Q3) and first quartiles (Q1) for each participant. Lower and upper bounds were determined as Q1 − 1.5 × IQR and Q3 + 1.5 × IQR, respectively. RTs falling outside of these bounds were excluded.

Eye-tracking data were analyzed manually. First, we verified that each participant had complete and usable recordings, including video, audio and eye-tracking data. Four dyads were excluded due to missing video, poor calibration of eye-tracking glasses, or incomplete recordings. Next, we calculated the number of gaze fixations directed towards the other participant during or immediately after stimulus presentation. The reference point for gaze fixation was established at the beginning of the experiment, when participants looked at each other before starting the task. Only clear and sustained gaze fixations were counted, while brief glances and gaze shifts triggered by the other participant’s occasional vocalizations were excluded.

RT analyses included only correct responses. Since the raw RT data did not meet the assumption of homogeneity, all RT values were log transformed. Continuous variables were standardized, and categorical variables were effect coded. The R package *lme4* (Bates et al., 2015) was employed to fit mixed-effect models (LMM). Random intercepts for participants and items were included. Attempts to include random slopes for within-subject factors did not converge; therefore, a simpler random-effects structure was retained. Statistical significance of fixed effects was assessed using Type III analysis of variance, with Satterthwaite’s approximation for degrees of freedom for RTs (Kuznetsova et al., 2017) and Wald chi-square tests for accuracy (Fox & Weisberg, 2019).

First, we fitted a linear mixed-effect model with word frequency and word length as fixed effects to examine whether these lexical variables significantly affected RTs. Next, we fitted a second model to analyze RTs, including task type (SCT vs. CCT), word type (abstract social vs. concrete social; abstract nonsocial vs. concrete nonsocial), task condition (dual vs. individual), and their interactions included as fixed effects. Participants and items were included as random effects. Generalized linear mixed-effect models with the same fixed-effect and random-effect structure were used to analyze errors, agreement, and gaze fixation rates. A full analysis of agreement and gaze fixation rates is provided in the Supplementary Materials, Sections 2 and 3.

## Results

A total of 9,428 trials (90.7%) were included in the accuracy analysis, and 8,379 of these trials were retained for the RT analysis, yielding an overall error rate of 11.12%. Word frequency and word length did not have significant effects on either RTs or accuracy (see the Supplementary Materials for full model outputs). Type III analyses of variance were conducted to evaluate the effects of task, word type, condition, and their interactions on RTs and error rates (Tables 2 and 3).

**Table 2:** Type III analysis of variance table using Satterthwaite’s method for reaction times (RTs)

*Effect*	*SS*	*MS*	*df1*	*df2*	*F*	*p*
Task	1.070	1.070	1	8248.909	52.877	<.001
Word type	1.967	0.656	3	94.505	32.399	<.001
Condition	0.151	0.151	1	50.033	7.465	.009
Task × word type	1.607	0.536	3	8248.868	26.472	<.001
Task × condition	0.267	0.267	1	8223.745	13.215	<.001
Word type × condition	0.024	0.008	3	8224.596	0.397	.755
Task × word type × condition	0.284	0.095	3	8223.962	4.684	.003

*SS* = sum of squares; *MS* = mean square; *df1* = numerator degrees of freedom; *df2* = denominator degrees of freedom

**Table 3:** Type III analysis of deviance using Wald chi-square tests for accuracy

*Effect*	χ^2^	*df*	*p*
Intercept	61.408	1	<.001
Task	0.082	1	.774
Word type	45.319	3	<.001
Condition	0.027	1	.869
Task × word type	2.331	3	.507
Task × condition	0.212	1	.645
Word type × condition	23.136	3	<.001
Task × word type × condition	11.815	3	.008

χ^2^ = chi-square value; *df* = degrees of freedom

**Fig. 2:**
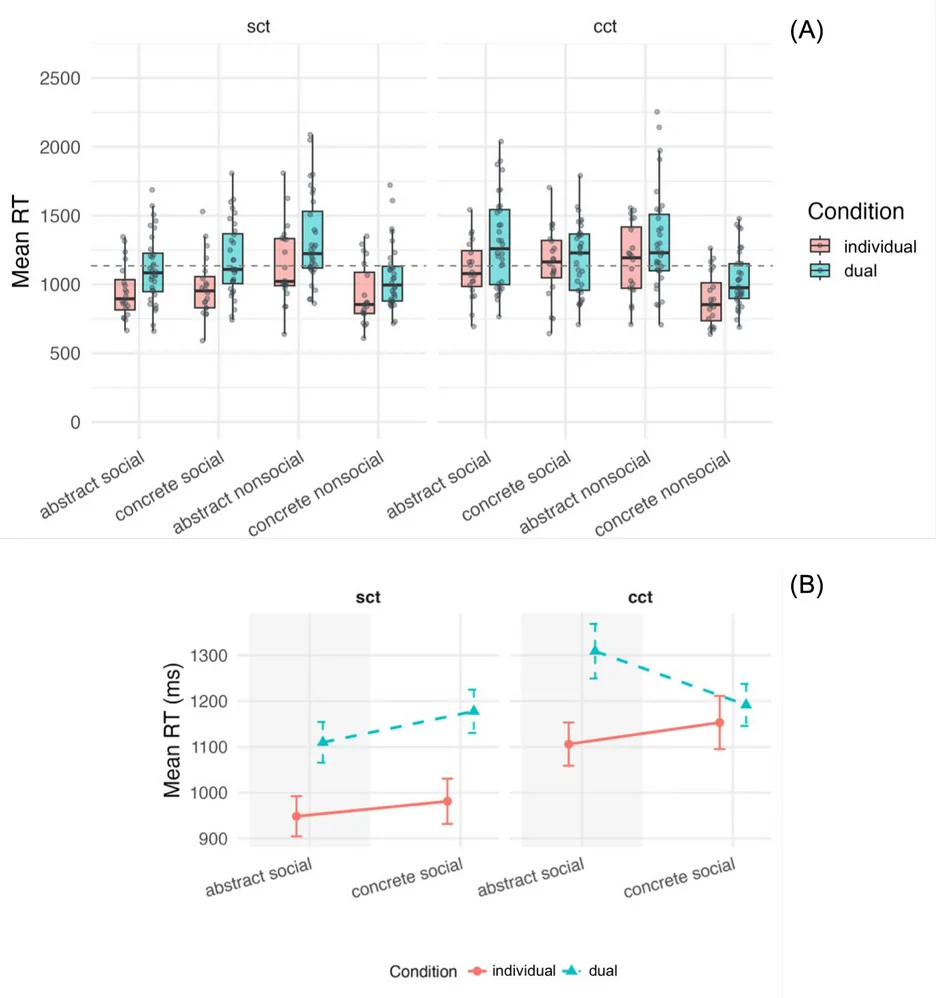
Interaction effects in RT: across all word types **(A)**, focus on social words **(B)**. The *y*-axis represents the mean reaction time across participants per word type (*x*-axis), task and condition. The dashed horizontal line (A) shows the overall mean Reaction Time across all conditions. sct = Socialness Categorization Task; cct = Concreteness Categorization Task. Error bars represent ±1 standard error (*SE*) of the mean. (Color figure online)

**Fig. 3:**
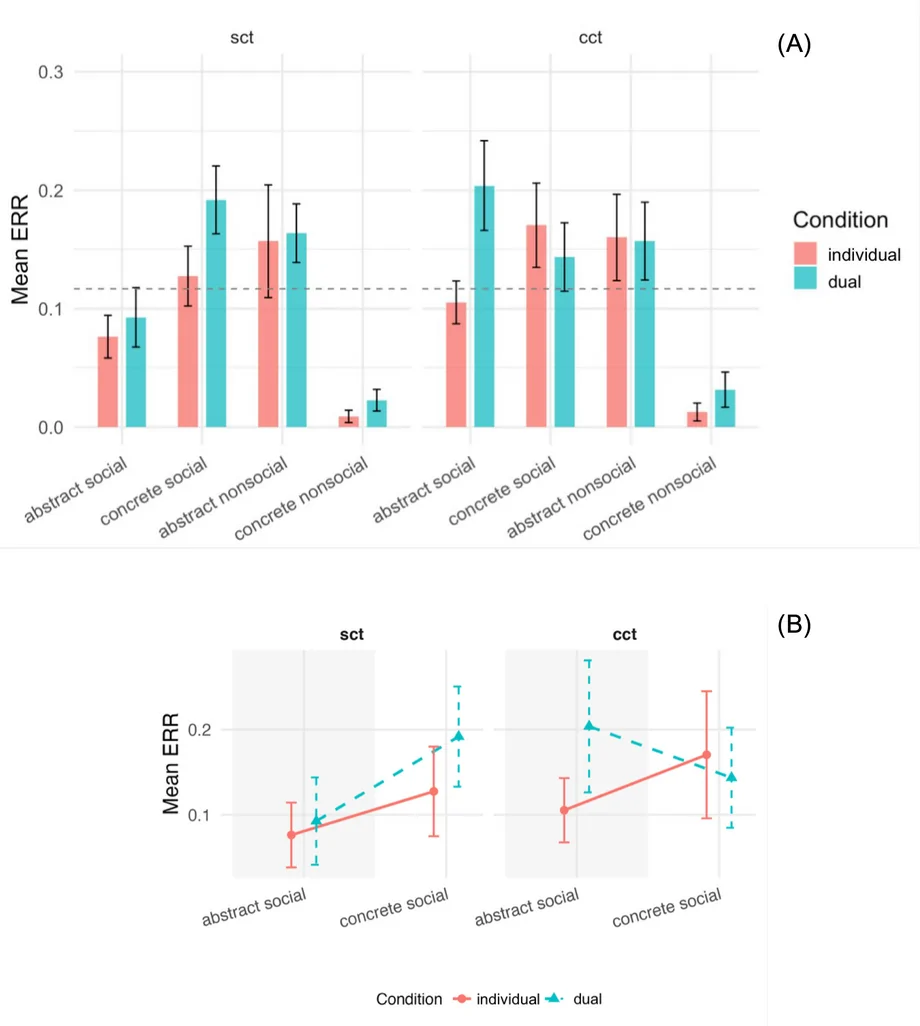
Interaction effects in error rates: across all word types **(A)**, focus on social words **(B)**. The *y*-axis represents the mean proportion of errors across participants per word type (*x*-axis). The dashed horizontal line (A) shows the overall mean error rate across all conditions. sct = Socialness Categorization Task; cct = Concreteness Categorization Task. Error bars indicate ±1 *SE*. (Color figure online)

The RT analysis revealed significant two-way interactions between task and word type, task and condition, as well as significant three-way interaction between task, word type, and condition. The accuracy analysis showed significant two-way interactions between word type and condition, along with significant three-way interaction between task, word type, and condition. These interaction effects are illustrated in Figs. 2 and 3.

To clarify the nature of these effects, we conducted a series of Estimated Marginal Means (EMMs) analyses with pairwise comparisons. These analyses contrasted word categories based on their socialness for each task and condition, as well as conditions for each task and word type. Full EMMs tables are provided in the Supplementary Materials (Section 1).

Overall, participants responded more quickly in the individual condition, particularly in the SCT (*p* <.001), suggesting that making decisions based on the social relevance of the words took longer in a social setting.

Across both tasks, the most salient difference between social and nonsocial words was the absence of a general concreteness effect for social words. In contrast, nonsocial words showed a robust concreteness effect in both RTs and accuracy, independent of task and condition (*p* <.001). The effects in social words depended on the condition. In the RTs, a facilitatory effect of socialness on abstract words processing was observed in the SCT in the dual condition only (*β* = −0.03, *SE* = 0.01, *t* = −2.72, *p* =.042). In the CCT, by contrast, a marginal inhibitory effect of socialness on abstract words processing emerged in the dual condition (*β* = 0.03, *SE* = 0.01, *t* = 2.55, *p* =.068).

A similar pattern was found in accuracy. In the SCT, abstract social words were processed more accurately than concrete social words in the dual condition only (*β* = −0.83, *SE* = 0.26, *z* = −3.22, *p* =.008). In the CCT, participants made significantly more errors in abstract social words in the dual compared to the individual condition (*β* = −0.88, *SE* = 0.26, *z* = −3.35, *p* <.001).

Together, these patterns suggest that the processing of abstract words, particularly abstract social words, is influenced by the presence of an interactive context. When socialness is the relevant dimension, an interactive context facilitates the processing of abstract social words; however, when concreteness is the relevant dimension, the reverse pattern emerges, with the interactive context making it harder to decide whether a word is abstract or concrete.

To explore this surprising and interesting pattern further, and since we hypothesized that the observed effects may not be solely driven by the experimental condition (dual or individual) but may also reflect the degree of actual social engagement during the task, we analyzed eye-tracking data, using gaze fixations as an indicator for participants’ social engagement during the task.

### Influence of cooperative behavior on word processing

Based on qualitative differences in gaze fixations, participants in the dual condition were post hoc categorized as either cooperative or noncooperative. Cooperative participants were those whose number of gaze fixations towards their partner exceeded the median global gaze count, whereas noncooperative participants had a number of gaze fixations below this median.

Although all participants in the dual condition performed the same tasks in identical settings, their levels of social engagement varied substantially. We hypothesized that this variation in cooperative behavior could have an impact on the main findings, particularly the effects observed in abstract social words, which appeared especially sensitive to social context.

**Fig. 4:**
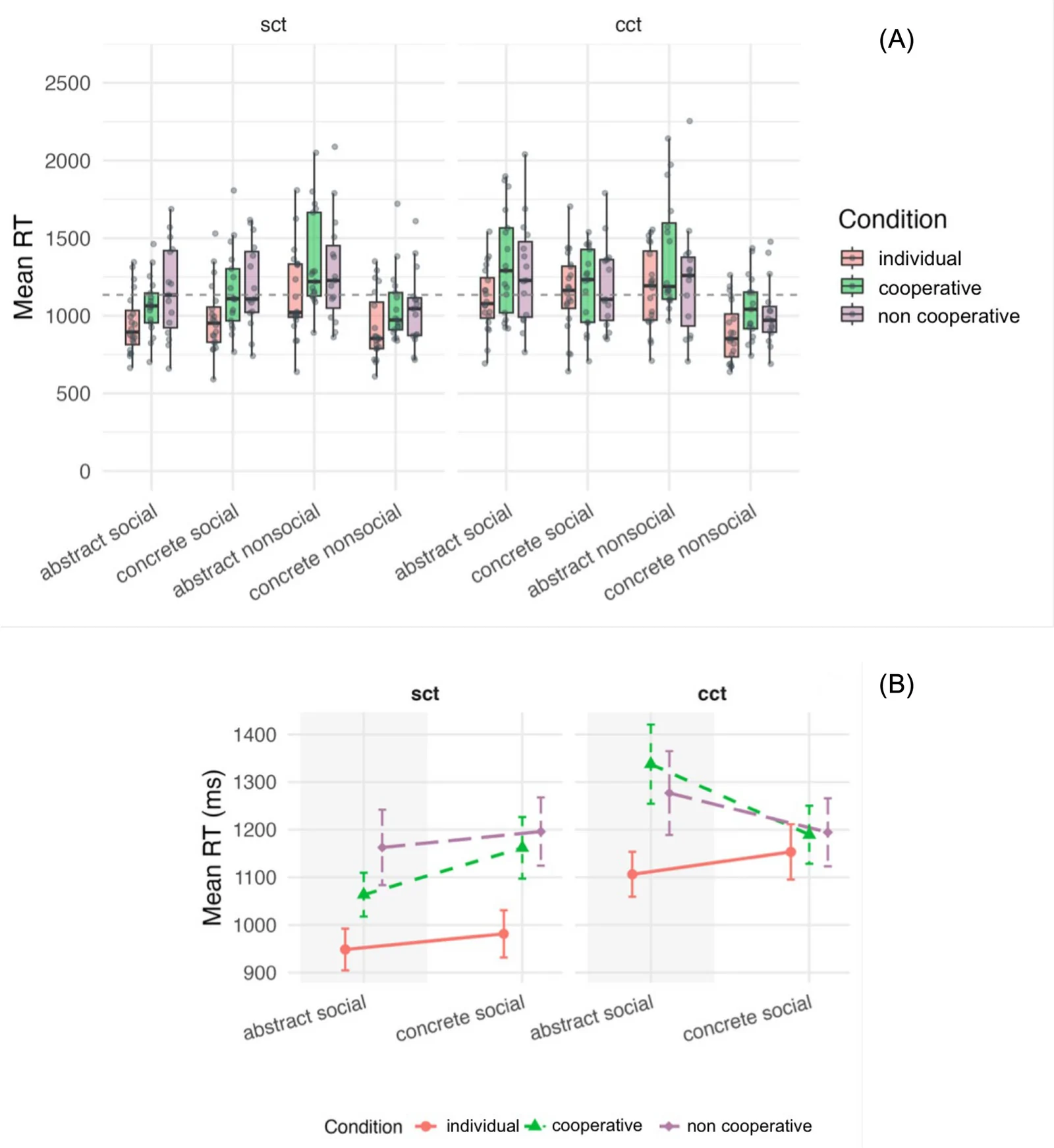
Interaction effects in RT: across all word types **(A)**, focus on social words **(B)**. The *y*-axis represents the mean reaction time across participants per word type (*x*-axis), task, and condition. The dashed horizontal line (A) shows the overall mean reaction time across all conditions. sct = Socialness Categorization Task; cct = Concreteness Categorization Task. Error bars represent ±1 standard error (*SE*) of the mean. (Color figure online)

**Fig. 5:**
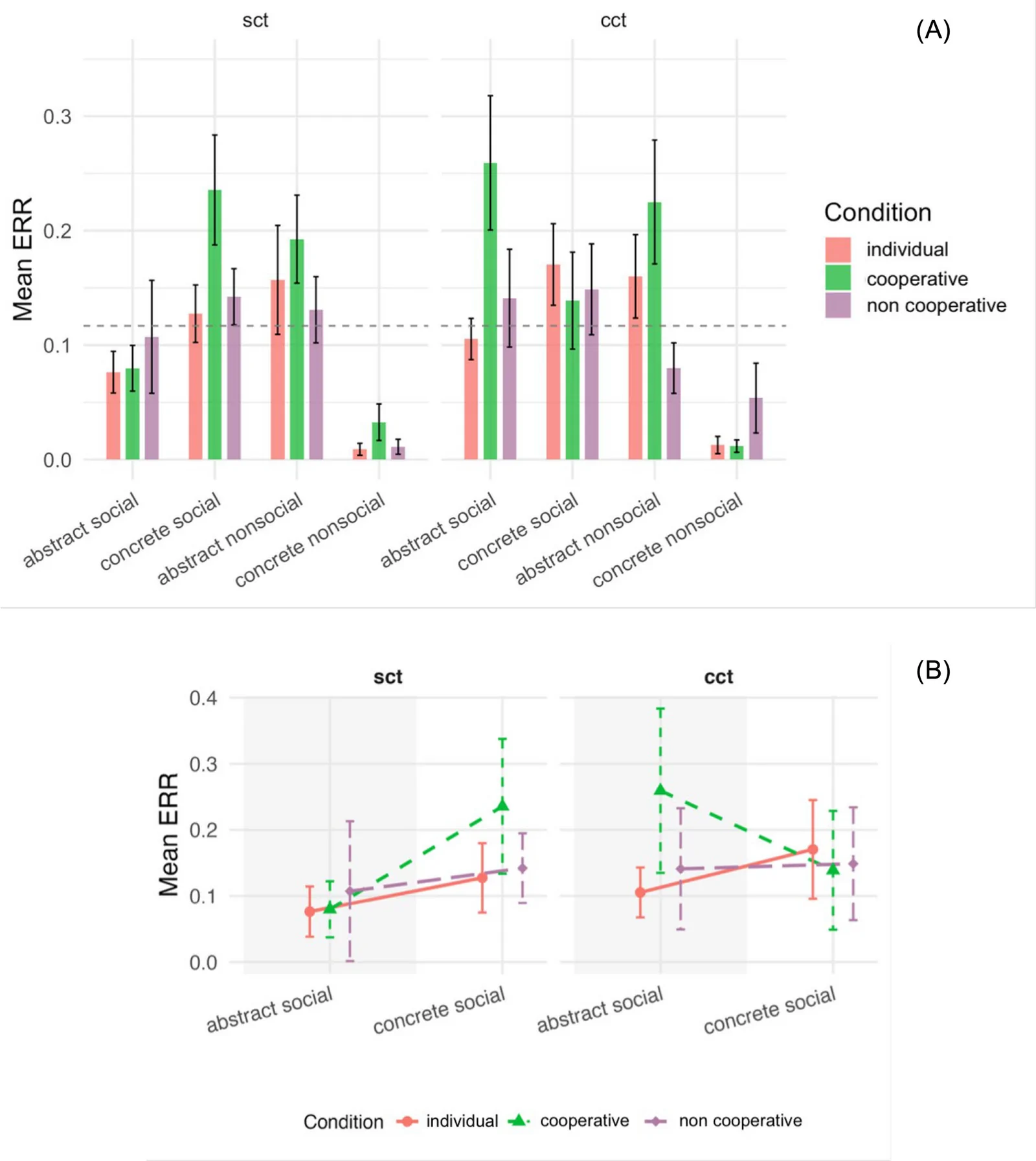
Interaction effects in accuracy: across all word types **(A)**, focus on social words **(B)**. The *y*-axis represents the mean proportion of errors across participants per word type (*x*-axis). The dashed horizontal line (A) shows the overall mean error rate across all conditions. sct = Socialness Categorization Task; cct = Concreteness Categorization Task. Error bars indicate ±1 *SE*. (Color figure online)

We therefore reanalyzed the data, comparing cooperative (*n* = 17) and noncooperative (*n* = 15) participants and the individual condition (*n* = 20). This additional analysis allowed us to assess the extent to which social engagement contributed to the observed effects in the interactive setup. At the dyad level, most pairs were homogeneous in their cooperative behavior (*n* = 13), with only a small number of mixed dyads (*n* = 3), limiting our ability to examine how dyad composition may modulate these effects. Results of these models and detailed pairwise comparisons are provided in the Supplementary Materials (Section 4). Figures 4 and 5 illustrate interaction effects in RTs and accuracy across all three conditions.

For the RTs, in the CCT, abstract social words were processed more slowly than concrete social words, but only in the cooperative condition (*β* = 0.05, *SE* = 0.02, *t* = 3.06, *p* =.014). Accuracy results showed that in the SCT, abstract social words were processed with fewer errors than concrete social words, again only in the cooperative group (*β* = −1.19, *SE* = 0.308, *t* = −3.88, *p* =.001). In contrast, in the CCT, abstract social words were processed with more errors than concrete social words, also only in the cooperative condition (*β* = 1.01, *SE* = 0.29, *t* = 3.47, *p* =.003).

Importantly, the effects for nonsocial words remained consistent with the main analysis: concrete nonsocial words were processed faster and more accurately than abstract nonsocial words, regardless of cooperative behavior.

This pattern was also reflected in gaze fixations. Across both tasks, abstract nonsocial words elicited more gaze fixations than concrete nonsocial words. In the CCT, however, abstract social words elicited more gaze fixations than concrete social words (*β* = 0.58, *SE* = 0.21, *z* = 2.72, *p* =.034). Moreover, abstract social words elicited more gaze fixations in the CCT than in the SCT (*β* = 0.72, *SE* = 0.16, *z* = 4.43, *p* <.0001).

Together, these findings indicate that the key effects observed for abstract social words, particularly in accuracy in the task where socialness was implicit (CCT), emerged only in the cooperative condition. This highlights a crucial role of social engagement in the processing of abstract social words and suggests that the previously reported effects were not only a consequence of performing the task in the interactive setting but also affected by participants’ actual engagement with their partner.

## Discussion

In this study, we investigated whether and how social interaction influences language processing. Participants completed two semantic categorization tasks in either an interactive (dual) or individual setting: a Socialness Categorization Task (SCT; social vs. nonsocial) and a Concreteness Categorization Task (CCT; concrete vs. abstract). The results show that words with social meaning are processed strikingly different depending on whether individuals are alone or interacting with another person. In the SCT, abstract social words were processed faster and more accurately than concrete social words, but only in the dual condition. In contrast, in the CCT, abstract social words were processed less accurately in the dual than in the individual condition. A post hoc analysis classifying dyads as cooperative or noncooperative based on gaze fixation revealed that these effects were driven not only by interaction, but also by social engagement. Specifically, cooperative participants processed abstract social words more accurately than concrete social words in the SCT, but more slowly and less accurately in the CCT. In other words, while in an isolated setting the data replicate Goriachun et al. (2025) in that socialness does not affect the processing of abstract words, in an interactive context and for cooperative persons the data provide evidence for the social grounding of abstract words.

While the facilitatory effects observed in the SCT confirm our initial hypothesis that an interactive setting and behavior enhances the processing of abstract social words, the disadvantage observed in the CCT was unexpected. However, a closer look at what happens in the CCT reveals a pattern consistent with the idea that social interaction makes abstract social words less abstract. Specifically, if interactive contexts enhance socially grounded interpretations, this may shift how abstract social words are categorized, increasing their likelihood of being judged as concrete in interactive settings. As a result, participants hesitate more (increase in RTs) and more often misclassify (increase in errors) abstract social words as concrete. Importantly, this should not be taken to imply that social interaction makes abstract words inherently more concrete. Rather, it suggests that interactive settings promote richer, socially grounded contextualization, which can shift the boundary between abstract and concrete dimensions depending on task demands.

This pattern cannot be attributed to the higher polysemy of abstract words in general, which makes them inherently more ambiguous and uncertain (Borghi, 2020; Mazzuca et al., 2024; Villani et al., 2022). Indeed, the social abstract words were processed with very high accuracy in both the noncooperative and individual conditions (*M*_acc_ = 0.96). It is only in the cooperative condition that accuracy dropped remarkably (*M*_acc_ = 0.61). Hence, in an interactive setting, and especially for cooperative participants, it seems that attempts to infer how a partner interprets a word may activate richer social experiences associated with abstract words, thereby enhancing their concreteness. This effect may not be solely driven by the interactive setting but may also reflect the engagement of social-cognitive mechanisms, such as perspective-taking or mentalizing, which are amplified when decisions are made jointly with a partner. However, the social metacognition implemented in the present paradigm is relatively generic: participants share a social context but do not explicitly negotiate word meaning. In this sense, the present design primarily captures how social context recruits social-cognitive processes that enhance the socialness features of word representations, rather than processes of shared meaning-making per se. This may explain why the effect is limited to social abstract words. More explicit forms of social interaction (e.g., discussing meanings) could promote shared representations even for nonsocial abstract words, whereas the general social context here enhances the grounding of words that have strong social features.

In sum, by demonstrating that both an interactive setting and cooperative behavior alters the processing of abstract social words, our study provides compelling empirical evidence for the hypothesis that understanding abstract concepts is facilitated by the input of others (Fini et al., 2021), and for theoretical accounts emphasizing the importance of social metacognition for grounding abstract knowledge (e.g., Borghi et al., 2021, 2025). This perspective further aligns with evidence that the interpretation of abstract words dynamically fluctuates with social context and interlocutor, increasing overlap between abstract and concrete representations in meaning-relevant contexts (Kewenig et al., 2024).

More generally, and in line with recent work by Kerr et al. (2025), this study demonstrates that word meaning is processed remarkably different in individual versus social contexts. The latter highlights the importance for paradigms that include social interaction when studying language and cognition and opens avenues for future research. For instance, as a first study to examine abstract meaning processing in dyads, it remains unclear whether the observed effects are specific to social words or extend to other semantic dimensions, such as emotional valence, which has also been suggested to play a central role in the grounding of abstract concepts (Kousta et al., 2011; Vigliocco et al., 2014).

Moreover, the way we measured social interaction and engagement captures only a fraction of the complexity of social cognition. Future research with other abstract word types and more ecological paradigms investigating multimodal alignment for instance could further expand our understanding how (abstract) meaning is jointly constructed in the social brain.

## Supplementary Information

Below is the link to the electronic supplementary material.

ESM 1 (DOCX 421 KB)

## Data Availability

The data supporting this article, including Supplementary Materials, are available at online repository (https://osf.io/38mnx/overview?view_only=ae2b4d8ef5264b459b84b99fa0b28387).

## Footnotes

1 Labvanced (https://www.labvanced.com)

## Code availability

All analysis and visualization R-scripts are available at online repository (https://osf.io/38mnx/overview?view_only=ae2b4d8ef5264b459b84b99fa0b28387).
