# Supplementary material for: The impact of social interaction on abstract concepts
Source: Psychon Bull Rev. 2026 Jun 18;33(6):178. doi: 10.3758/s13423-026-02941-4 (PMC13279488; doi:10.3758/s13423-026-02941-4)
Supplement: Supplementary file 1 — (DOCX 421 KB) [file 13423_2026_2941_MOESM1_ESM.docx]

# Section 1: Mixed models and pairwise comparisons for the main analysis

Type III Analysis of Variance (Satterthwaite’s Method) for Reaction Times

| **Predictor** | **Sum Sq** | **Mean Sq** | **Num df** | **Den df** | **F** | **p** |
| --- | --- | --- | --- | --- | --- | --- |
| Frequency | 0.000 | 0.000 | 1 | 96.6 | 0.01 | .931 |
| Length | 0.007 | 0.007 | 1 | 95.7 | 0.35 | .557 |
| Trial | 0.026 | 0.026 | 1 | 96.1 | 1.29 | .259 |
| Task | 0.938 | 0.938 | 1 | 8240.8 | 45.89 | < .001 *** |
| Interaction Status | 0.153 | 0.153 | 1 | 50.0 | 7.50 | .009 ** |
| Trial × Interaction Status | 0.097 | 0.097 | 1 | 8227.4 | 4.77 | .029 * |
| Trial × Task | 0.014 | 0.014 | 1 | 8241.8 | 0.66 | .415 |
| Task × Interaction Status | 0.256 | 0.256 | 1 | 8228.2 | 12.54 | < .001 *** |
| Trial × Task × Interaction Status | 0.031 | 0.031 | 1 | 8228.5 | 1.52 | .217 |

Type III Analysis of Deviance (Chi-square Tests) for Accuracy

| **Predictor** | **χ²** | **df** | **p** |
| --- | --- | --- | --- |
| Frequency | 2.30 | 1 | .129 |
| Length | 1.27 | 1 | .259 |
| Trial | 2.61 | 1 | .106 |
| Task | 5.63 | 1 | .018 * |
| Interaction Status | 1.39 | 1 | .238 |
| Trial × Interaction Status | 1.14 | 1 | .286 |
| Trial × Task | 0.00 | 1 | .971 |
| Task × Interaction Status | 0.46 | 1 | .497 |
| Trial × Task × Interaction Status | 1.03 | 1 | .310 |

**Pairwise comparisons**

**Reaction Time**

| **Contrast** | **Task** | **Condition** | **β** | **SE** | **t** | **p** |
| --- | --- | --- | --- | --- | --- | --- |
| abstract social - concrete social | sct | baseline | -0.013 | 0.013 | -0.98 | 1.000 |
| abstract social - concrete social | sct | dual | -0.032 | 0.012 | -2.72 | 0.042 |
| abstract social - concrete social | cct | baseline | -0.003 | 0.013 | -0.24 | 1.000 |
| abstract social - concrete social | cct | dual | 0.031 | 0.012 | 2.55 | 0.068 |
| abstract nonsocial - concrete nonsocial | sct | baseline | 0.080 | 0.013 | 6.20 | <0.001 |
| abstract nonsocial - concrete nonsocial | sct | dual | 0.094 | 0.012 | 8.06 | <0.001 |
| abstract nonsocial - concrete nonsocial | cct | baseline | 0.112 | 0.013 | 8.58 | <0.001 |
| abstract nonsocial - concrete nonsocial | cct | dual | 0.090 | 0.012 | 7.68 | <0.001 |

| **Contrast** | **Task** | **Type** | **β** | **SE** | **t** | **p** |
| --- | --- | --- | --- | --- | --- | --- |
| baseline - dual | sct | abstract nonsocial | -0.071 | 0.022 | -3.25 | 0.002 |
| baseline - dual | sct | abstract social | -0.059 | 0.022 | -2.72 | 0.008 |
| baseline - dual | sct | concrete nonsocial | -0.057 | 0.021 | -2.68 | 0.009 |
| baseline - dual | sct | concrete social | -0.078 | 0.022 | -3.58 | <0.001 |
| baseline - dual | cct | abstract nonsocial | -0.037 | 0.022 | -1.70 | 0.093 |
| baseline - dual | cct | abstract social | -0.055 | 0.022 | -2.50 | 0.015 |
| baseline - dual | cct | concrete nonsocial | -0.059 | 0.021 | -2.77 | 0.007 |
| baseline - dual | cct | concrete social | -0.021 | 0.022 | -0.94 | 0.350 |

**Accuracy**

| **Contrast** | **Task** | **Condition** | **β** | **SE** | **z** | **p** |
| --- | --- | --- | --- | --- | --- | --- |
| abstract social - concrete social | sct | baseline | -0.608 | 0.305 | -2.00 | 0.276 |
| abstract social - concrete social | sct | dual | -0.834 | 0.259 | -3.22 | 0.008 |
| abstract social - concrete social | cct | baseline | -0.614 | 0.286 | -2.15 | 0.191 |
| abstract social - concrete social | cct | dual | 0.484 | 0.250 | 1.94 | 0.318 |
| abstract nonsocial - concrete nonsocial | sct | baseline | 3.276 | 0.544 | 6.02 | <0.001 |
| abstract nonsocial - concrete nonsocial | sct | dual | 2.259 | 0.332 | 6.81 | <0.001 |
| abstract nonsocial - concrete nonsocial | cct | baseline | 2.952 | 0.468 | 6.31 | <0.001 |
| abstract nonsocial - concrete nonsocial | cct | dual | 1.947 | 0.316 | 6.16 | <0.001 |

| **Contrast** | **Task** | **Type** | **β** | **SE** | **z** | **p** |
| --- | --- | --- | --- | --- | --- | --- |
| baseline - dual | sct | abstract nonsocial | -0.071 | 0.249 | -0.28 | 0.777 |
| baseline - dual | sct | abstract social | -0.308 | 0.288 | -1.07 | 0.284 |
| baseline - dual | sct | concrete nonsocial | -1.088 | 0.573 | -1.90 | 0.058 |
| baseline - dual | sct | concrete social | -0.534 | 0.255 | -2.10 | 0.036 |
| baseline - dual | cct | abstract nonsocial | 0.042 | 0.251 | 0.17 | 0.869 |
| baseline - dual | cct | abstract social | -0.881 | 0.263 | -3.35 | <0.001 |
| baseline - dual | cct | concrete nonsocial | -0.963 | 0.490 | -1.96 | 0.049 |
| baseline - dual | cct | concrete social | 0.217 | 0.254 | 0.86 | 0.392 |

Section 2: Agreement rates analysis

The analysis of agreement rates revealed a significant interaction between task and word type (Figure 1). In the SCT, participants demonstrated higher agreement rates for abstract social words compared to concrete social words (b = 0.69, SE = 0.26, z = 2.71, p = 0.034). However, no significant differences in agreement rates were observed between these word categories in the CCT.

Comparing agreement rates across tasks, abstract social words had higher agreement rates in the SCT compared to the CCT (b = 0.62, SE = 0.15, z = 4.14, p < .0001). Conversely, abstract nonsocial words had higher agreement rates in the CCT compared to the SCT (*b* = 0.44, *SE* = 0.140, *z* = 3.149, *p* = 0.002). Concrete social words also had higher agreement rates in the CCT than in the SCT (b = 0.3, *SE* = 0.14, *z* = 2.14, *p* = 0.033).

Table 1. Analysis of Deviance Table (Type III Wald chi-square tests) for Agreement Rates

| *Effect* | *Chisq* | *df* | *P-Value* |
| --- | --- | --- | --- |
| Intercept | 83.816 | 1 | < .001 |
| Task | 9.917 | 1 | 0.002 |
| Word Type | 46.612 | 3 | < .001 |
| Task x Word Type | 31.514 | 3 | < .001 |

Figure 1. Interaction effects in Agreement Rates in dual condition

**
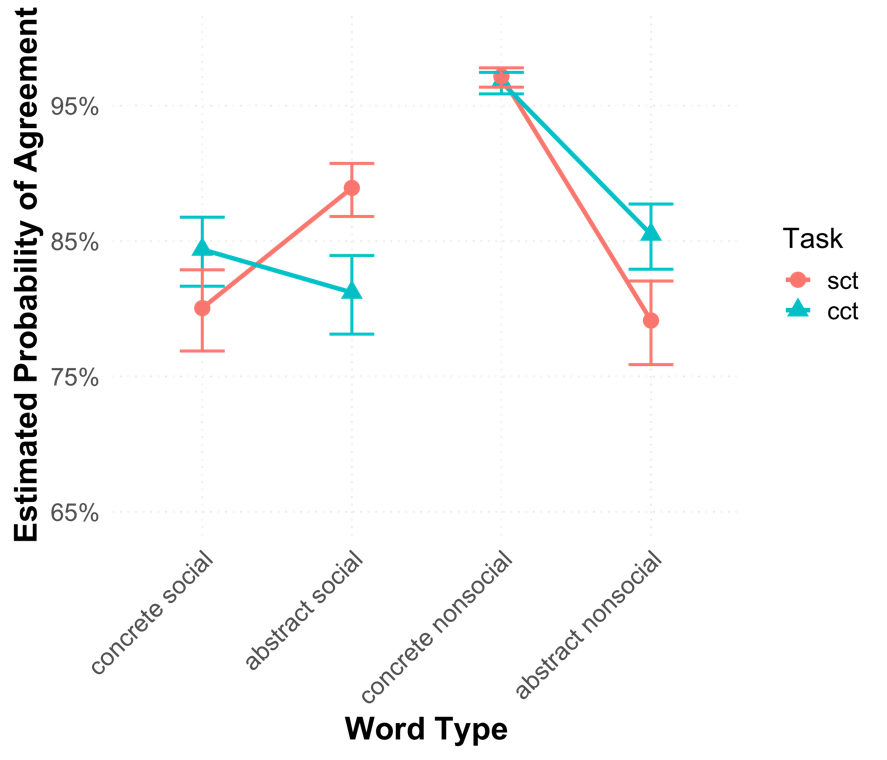
**

Section 3: Gaze fixation rates analysis

The analysis of gaze fixation rate revealed a significant interaction between task and word type (Figure 4). Across both tasks, abstract nonsocial words elicited more gaze fixations than concrete nonsocial words (SCT: b = 1.06, SE = 0.22, z = 4.75, p < .0001; CCT: b = 1.68, SE = 0.24, z = 6.94, p < .0001). However, gaze fixation differences between abstract social and concrete social words varied by tasks. While no significant difference was found in the SCT, in the CCT, abstract social words elicited more gaze fixations than concrete social words (b = 0.58, SE = 0.21, z = 2.72, p = 0.034).

Figure 2. Individual gaze fixation rates illustrating cooperative and noncooperative group separation


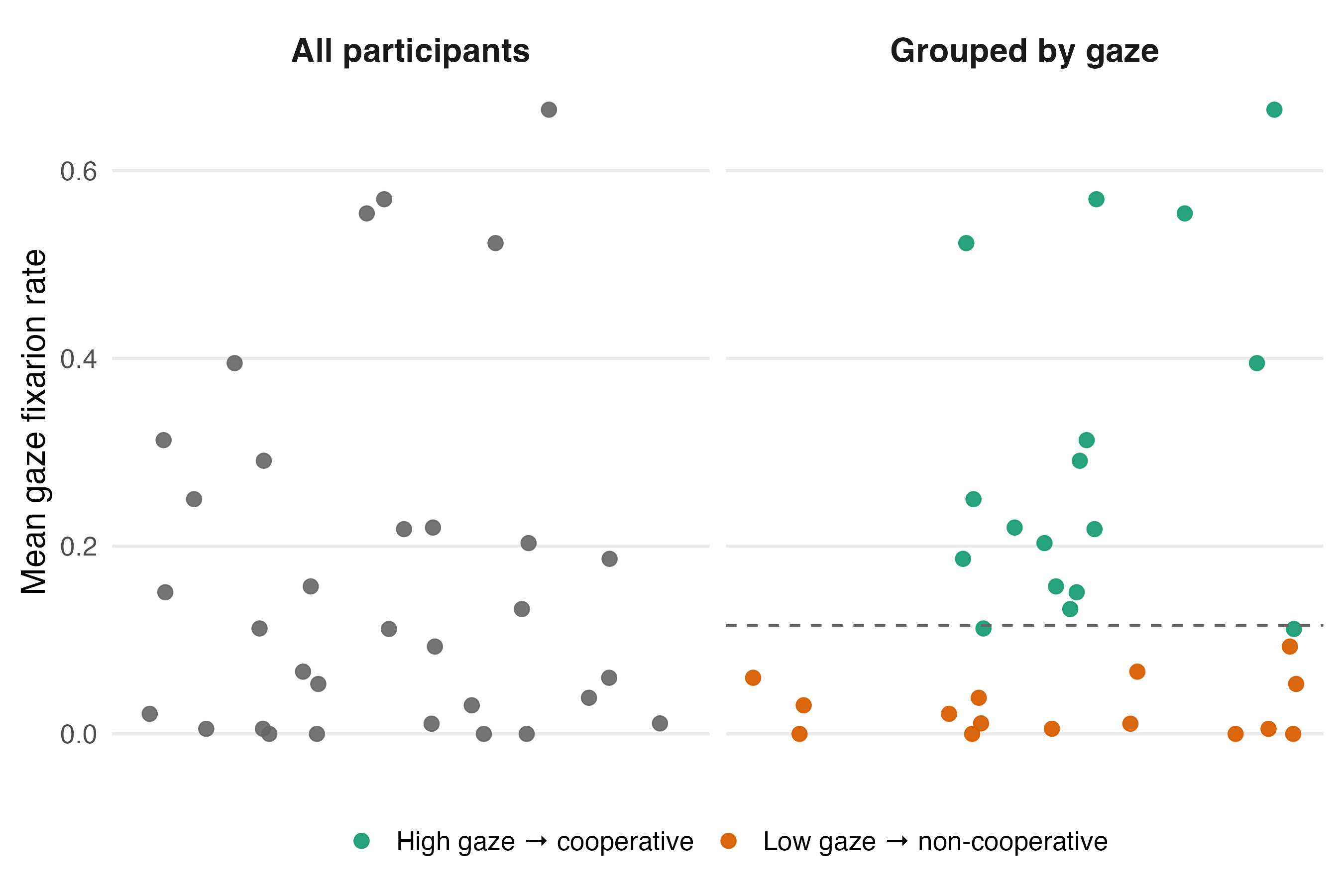


The effect of task was significant in abstract social words, which elicited more gaze fixations in the CCT compared to the SCT (b = 0.72, SE = 0.16, z = 4.43, p < .0001). Conversely, for concrete nonsocial words, more gaze fixations were observed in SCT than in CCT (b = 0.54, SE = 0.21, z = 2.52, p = 0.012).

Table 2. Analysis of Deviance Table (Type III Wald chi-square tests) for Gaze Fixation Rates

| *Effect* | *Chisq* | *df* | *P-Value* |
| --- | --- | --- | --- |
| Intercept | 30.459 | 1 | < .001 |
| Task | 0.243 | 1 | 0.622 |
| Type | 67.844 | 3 | < .001 |
| Task x Type | 27.436 | 3 | < .001 |

Figure 4. Interaction effects in Gaze fixation Rates in dual condition


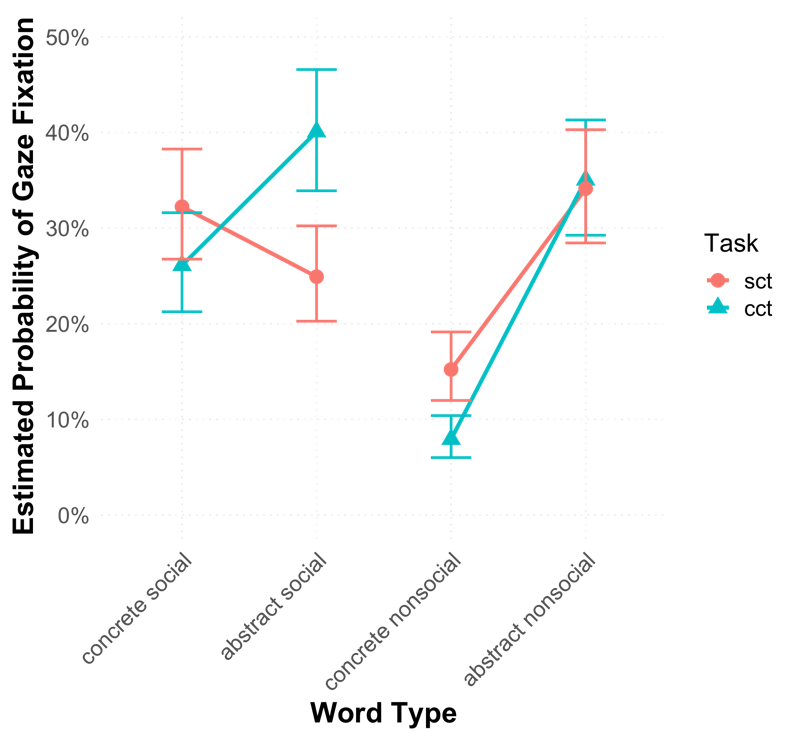


Section 4: Post-Hoc Analysis

**Reaction Time**

| **contrast** | **group** | **task** | **β** | **SE** | **t** | **p** |
| --- | --- | --- | --- | --- | --- | --- |
| abstract nonsocial - concrete nonsocial | baseline | cct | 0.112 | 0.013 | 8.587 | 0 |
| abstract nonsocial - concrete nonsocial | cooperative | cct | 0.113 | 0.014 | 7.889 | 0 |
| abstract nonsocial - concrete nonsocial | non_cooperative | cct | 0.072 | 0.014 | 5.166 | 0 |
| abstract nonsocial - concrete nonsocial | baseline | sct | 0.08 | 0.013 | 6.208 | 0 |
| abstract nonsocial - concrete nonsocial | cooperative | sct | 0.106 | 0.014 | 7.536 | 0 |
| abstract nonsocial - concrete nonsocial | non_cooperative | sct | 0.082 | 0.014 | 5.827 | 0 |
| abstract social - concrete social | baseline | cct | -0.003 | 0.013 | -0.243 | 1 |
| abstract social - concrete social | cooperative | cct | 0.046 | 0.015 | 3.06 | 0.014 |
| abstract social - concrete social | non_cooperative | cct | 0.016 | 0.015 | 1.081 | 1 |
| abstract social - concrete social | baseline | sct | -0.013 | 0.013 | -0.984 | 1 |
| abstract social - concrete social | cooperative | sct | -0.033 | 0.014 | -2.313 | 0.127 |
| abstract social - concrete social | non_cooperative | sct | -0.03 | 0.014 | -2.068 | 0.235 |

| **contrast** | **task** | **type** | **β** | **SE** | **t** | **p** |
| --- | --- | --- | --- | --- | --- | --- |
| baseline - cooperative | sct | abstract nonsocial | -0.079 | 0.025 | -3.102 | 0.008 |
| baseline - noncooperative | sct | abstract nonsocial | -0.062 | 0.026 | -2.371 | 0.062 |
| cooperative - noncooperative | sct | abstract nonsocial | 0.017 | 0.027 | 0.617 | 1 |
| baseline - cooperative | sct | abstract social | -0.048 | 0.025 | -1.903 | 0.184 |
| baseline - noncooperative | sct | abstract social | -0.071 | 0.026 | -2.709 | 0.026 |
| cooperative - noncooperative | sct | abstract social | -0.023 | 0.027 | -0.837 | 1 |
| baseline - cooperative | sct | concrete nonsocial | -0.054 | 0.025 | -2.154 | 0.105 |
| baseline - noncooperative | sct | concrete nonsocial | -0.061 | 0.026 | -2.354 | 0.065 |
| cooperative - noncooperative | sct | concrete nonsocial | -0.007 | 0.027 | -0.253 | 1 |
| baseline - cooperative | sct | concrete social | -0.068 | 0.025 | -2.666 | 0.029 |
| baseline - noncooperative | sct | concrete social | -0.087 | 0.026 | -3.343 | 0.004 |
| cooperative - noncooperative | sct | concrete social | -0.02 | 0.027 | -0.717 | 1 |
| baseline - cooperative | cct | abstract nonsocial | -0.066 | 0.026 | -2.581 | 0.036 |
| baseline - noncooperative | cct | abstract nonsocial | -0.013 | 0.026 | -0.501 | 1 |
| cooperative - noncooperative | cct | abstract nonsocial | 0.053 | 0.027 | 1.944 | 0.168 |
| baseline - cooperative | cct | abstract social | -0.059 | 0.026 | -2.311 | 0.071 |
| baseline - noncooperative | cct | abstract social | -0.05 | 0.026 | -1.914 | 0.18 |
| cooperative - noncooperative | cct | abstract social | 0.009 | 0.028 | 0.333 | 1 |
| baseline - cooperative | cct | concrete nonsocial | -0.065 | 0.025 | -2.606 | 0.034 |
| baseline - noncooperative | cct | concrete nonsocial | -0.053 | 0.026 | -2.042 | 0.136 |
| cooperative - noncooperative | cct | concrete nonsocial | 0.012 | 0.027 | 0.461 | 1 |
| baseline - cooperative | cct | concrete social | -0.011 | 0.025 | -0.416 | 1 |
| baseline - noncooperative | cct | concrete social | -0.031 | 0.026 | -1.181 | 0.725 |
| cooperative - non_cooperative | cct | concrete social | -0.02 | 0.027 | -0.748 | 1 |

**Accuracy**

| **contrast** | **group** | **task** | **β** | **SE** | **z** | **p** |
| --- | --- | --- | --- | --- | --- | --- |
| abstract nonsocial - concrete nonsocial | baseline | cct | 2.951 | 0.469 | 6.291 | 0 |
| abstract social - concrete social | baseline | cct | -0.617 | 0.287 | -2.149 | 0.19 |
| abstract nonsocial - concrete nonsocial | cooperative | cct | 3.525 | 0.547 | 6.441 | 0 |
| abstract social - concrete social | cooperative | cct | 1.005 | 0.29 | 3.466 | 0.003 |
| abstract nonsocial - concrete nonsocial | non_cooperative | cct | 0.553 | 0.377 | 1.466 | 0.856 |
| abstract social - concrete social | non_cooperative | cct | -0.185 | 0.307 | -0.603 | 1 |
| abstract nonsocial - concrete nonsocial | baseline | sct | 3.274 | 0.545 | 6.007 | 0 |
| abstract social - concrete social | baseline | sct | -0.611 | 0.305 | -2 | 0.273 |
| abstract nonsocial - concrete nonsocial | cooperative | sct | 2.071 | 0.372 | 5.566 | 0 |
| abstract social - concrete social | cooperative | sct | -1.193 | 0.308 | -3.881 | 0.001 |
| abstract nonsocial - concrete nonsocial | non_cooperative | sct | 2.707 | 0.553 | 4.894 | 0 |
| abstract social - concrete social | non_cooperative | sct | -0.424 | 0.313 | -1.356 | 1 |

| **contrast** | **type** | **task** | **β** | **SE** | **z** | **p** |
| --- | --- | --- | --- | --- | --- | --- |
| baseline - cooperative | abstract nonsocial | cct | -0.494 | 0.278 | -1.778 | 0.226 |
| baseline - non_cooperative | abstract nonsocial | cct | 0.852 | 0.321 | 2.649 | 0.024 |
| cooperative - non_cooperative | abstract nonsocial | cct | 1.346 | 0.326 | 4.128 | 0 |
| baseline - cooperative | abstract social | cct | -1.299 | 0.29 | -4.485 | 0 |
| baseline - non_cooperative | abstract social | cct | -0.327 | 0.313 | -1.042 | 0.892 |
| cooperative - non_cooperative | abstract social | cct | 0.973 | 0.306 | 3.182 | 0.004 |
| baseline - cooperative | concrete nonsocial | cct | 0.08 | 0.664 | 0.12 | 1 |
| baseline - non_cooperative | concrete nonsocial | cct | -1.546 | 0.514 | -3.009 | 0.008 |
| cooperative - non_cooperative | concrete nonsocial | cct | -1.626 | 0.589 | -2.762 | 0.017 |
| baseline - cooperative | concrete social | cct | 0.323 | 0.295 | 1.095 | 0.821 |
| baseline - non_cooperative | concrete social | cct | 0.105 | 0.296 | 0.355 | 1 |
| cooperative - non_cooperative | concrete social | cct | -0.217 | 0.318 | -0.684 | 1 |
| baseline - cooperative | abstract nonsocial | sct | -0.302 | 0.28 | -1.08 | 0.84 |
| baseline - non_cooperative | abstract nonsocial | sct | 0.211 | 0.299 | 0.705 | 1 |
| cooperative - non_cooperative | abstract nonsocial | sct | 0.513 | 0.307 | 1.67 | 0.285 |
| baseline - cooperative | abstract social | sct | -0.183 | 0.333 | -0.549 | 1 |
| baseline - non_cooperative | abstract social | sct | -0.439 | 0.33 | -1.329 | 0.552 |
| cooperative - non_cooperative | abstract social | sct | -0.256 | 0.342 | -0.749 | 1 |
| baseline - cooperative | concrete nonsocial | sct | -1.505 | 0.597 | -2.52 | 0.035 |
| baseline - non_cooperative | concrete nonsocial | sct | -0.357 | 0.72 | -0.496 | 1 |
| cooperative - non_cooperative | concrete nonsocial | sct | 1.149 | 0.601 | 1.911 | 0.168 |
| baseline - cooperative | concrete social | sct | -0.765 | 0.283 | -2.699 | 0.021 |
| baseline - non_cooperative | concrete social | sct | -0.252 | 0.301 | -0.836 | 1 |
| cooperative - non_cooperative | concrete social | sct | 0.513 | 0.3 | 1.708 | 0.263 |

Section 5: Instructions for Participants

**Instruction for the SCT**

**Original**

Sur l'écran, des mots apparaîtront. Votre tâche consiste à déterminer si chaque mot décrit un concept lié ou non à une expérience sociale.

Bien que les catégories sémantiques soient subjectives, voici les directives à suivre pour décider à quelle catégorie appartient chaque mot.

Les concepts sociaux incluent :

* Les références à une personne ou à un groupe de personnes, ainsi que leurs caractéristiques sociales (par exemple, « ami », « tribu », « ethnicité »).

* Les comportements ou interactions sociales (par exemple, « coopération », « salutation »).

* Les rôles sociaux (par exemple, « scientifique »).

* Les espaces ou événements sociaux (par exemple, « bar », « concours »).

* Les institutions sociales (par exemple, « hôpital ») ou les systèmes (par exemple, « nation »).

* Les valeurs (par exemple, « sincérité ») ou idéologies (par exemple, « féminisme »).

Les concepts non sociaux sont tous les autres mots qui ne sont pas liés à l'expérience sociale :

* Les objets (par exemple, « vase », « fenêtre »).

* Les êtres vivants (par exemple, « éléphant »).

* Les qualités (par exemple, « efficacité », « proportion »).

* Les processus (par exemple, « conséquence », « réaction »), y compris certains processus mentaux (par exemple, « perception »).

**English Translation**

Words will appear on the screen. Your task is to determine whether each word describes a concept related to a social experience or not.

Although semantic categories are subjective, please follow these guidelines to decide which category each word belongs to.

Social concepts include:

* References to a person or a group of people, as well as their social characteristics (e.g., “friend”, “tribe”, “ethnicity”)

* Social behaviors or interactions (e.g., “cooperation”, “greeting”)

* Social roles (e.g., “scientist”)

* Social spaces or events (e.g., “bar”, “competition”)

* Social institutions (e.g., “hospital”) or systems (e.g., “nation”)

* Values (e.g., “sincerity”) or ideologies (e.g., “feminism”)

Non-social concepts include all other words not related to social experience:

* Objects (e.g., “vase”, “window”)

* Living beings (e.g., “elephant”)

* Qualities (e.g., “efficiency”, “proportion”)

* Processes (e.g., “consequence”, “reaction”), including some mental processes (e.g., “perception”)

**Instruction for the CCT**

**Original**

Sur l'écran, des mots vont apparaître. Votre tâche consiste à décider si chaque mot est abstrait ou concret.

Bien que les catégories sémantiques soient subjectives, voici les directives pour déterminer à quelle catégorie appartient chaque mot.

Mots concrets :

* Objets tangibles et visibles (par exemple, "voiture", "carré")

* Êtres vivants (par exemple, "cheval")

* Personnes (par exemple, "infirmière")

* Choses pouvant être perçues avec vos cinq sens (par exemple, "aboiement", "pluie", "chauffage")

Mots abstraits :

* Objets non tangibles qui ne peuvent pas être perçus avec vos cinq sens (par exemple, "idée", "croyance")

* Mots décrivant des processus mentaux (par exemple, "interprétation", "raisonnement")

* États (par exemple, "paix", "déni")

* Qualités (par exemple, "curiosité", "modernité")

**English Translation**

Words will appear on the screen. Your task is to decide whether each word is abstract or concrete.

Although semantic categories are subjective, please follow these guidelines to determine which category each word belongs to.

Concrete words:

* Tangible and visible objects (e.g., “car”, “square”)

* Living beings (e.g., “horse”)

* People (e.g., “nurse”)

* Things that can be perceived with your five senses (e.g., “barking”, “rain”, “heating”)

Abstract words:

* Intangible entities that cannot be perceived with your five senses (e.g., “idea”, “belief”)

* Words describing mental processes (e.g., “interpretation”, “reasoning”)

* States (e.g., “peace”, “denial”)

* Qualities (e.g., “curiosity”, “modernity”)
